# A293 IS NON AL C OHOLIC FATTY PANCREATIC DISEASESE IS PATHOLOGICAL DISEASESE FOR DEVELOPMENT OF DIABETES :AN EGYPTIAN PILOT STUDY

**DOI:** 10.1093/jcag/gwac036.293

**Published:** 2023-03-07

**Authors:** R M Elbadawy, M S Natboli, G M Masoud

**Affiliations:** 1 Gastroenterology, Hepatology, Banha University, Banha; 2 Biochemistry, Ain Shams University, Cairo, Egypt

## Abstract

**Background:**

Non Alcoholic Fatty Pancreatic disease(NAFPD), Fatty pancreas emerged as health problem world wide parralel to the epidemic of obesity .NAFPD lead chronic pancreatitis ,exocrine pancreatic insufficiency as per research proved . NAFPD also can lead to Diabeties Mellites (DM ) and lasly pancreatic adenocarcinoma . As per UEG cancer pancreas will be the the 3rd cancer world wide which is so serious and almost most of the patients come late with irriversible stage . Of sure most the patients with cancer pancreas have DM and other pancreatic disorders .

**Purpose:**

The Aim of the research was to explore that NAFPD is the first stage in the development of DM

**Method:**

Eighty -Eight subjects was inculded in the study both male and female ,DM or non DM and obese or not obese with mean age 25-66 years old .The subjects were divided into 4 groups .Group (1)DM subjects withnormal body mass index<25kg/m^2^. Group (2)Subjects DM with body mass index >25 kg/m^2^ obese .Group (3)subjects nonDM ,non obese with normal body mass index.Group (4)subjects non DM withbody mass index >25kg/m^2 . ^ Each group 22 subjects both male and females.All routine investigations were done inculding lipid profile , liver functions .Nafpd was diagnosed by abdominal ultrasound ,Toshiba using convex probecompared to right kidney , liver and brightness of diaphragm .The echogencity was graded from (0-3 )..statistical analysis by SPSS version 22 was used.

**Result(s):**

Mean age was 44.08+_12.4 years old for all subjects. Mean Body Mass Index(BMI) was 29.73_+8.15 kg/m^2^ .Male was 73% while female 27% of NAFPD diagnosis .NAFPD was present in non obese non DM as follow ,grade 1,2,3(22.7%,27.3% and 22.7% respectively ).While NAFPD was present in DM non obese as follow in grades 1.2,3(22.7%,31.8% and45.4% respectively ) means that there was increas in % of DM development especially grade 2,3 . NAFPD was in obese non DM as follow in grades 2,3(31.8%,68.2% respectively . While in in DM obese NAFPD was present as follow (18.2%,81.2%). .This means that there was increaseincrease in % of DM development.

**Conclusion(s):**

NAFPD proved to be the first step in development of DM , it is an alert sign for health insitiute to care the issue to prevent DM from the start .

Aknowledgment to Academic Scientific Research & Technology for funding jesor project no 5269 , as this work part from the project

**Please acknowledge all funding agencies by checking the applicable boxes below:**

None

**Disclosure of Interest:**

R. Elbadawy Grant / Research support from: Aknowldgement to Academic Scientific Research & Technology , Egypt (ASRT ) for funding jesor project as this work part of the project, Paid Instructor of: 1000000 egyptian pound, M. Natboli: None Declared, G. Masoud: None Declared

